# Yoga Therapy in Children with Cystic Fibrosis Decreases Immediate Anxiety and Joint Pain

**DOI:** 10.1155/2016/9429504

**Published:** 2016-12-19

**Authors:** Christopher McNamara, Mahrya Johnson, Lisa Read, Heidi Vander Velden, Megan Thygeson, Meixia Liu, Laura Gandrud, John McNamara

**Affiliations:** ^1^Children's Respiratory and Critical Care Specialists, PA, Minneapolis, MN, USA; ^2^Children's Hospitals and Clinics of Minnesota, Minneapolis, MN, USA; ^3^Department of Pediatrics, University of Minnesota, Minneapolis, MN, USA

## Abstract

This study was designed to determine whether yoga might alleviate symptoms of pain, sleep disturbance, anxiety, and depression in children with cystic fibrosis (CF). CF is the most common genetic, life-limiting chronic disease among Caucasian populations. It primarily affects the lungs but also many other secretory organs and consequently leads to significant morbidities. Research has shown that children with CF have significantly increased depression, anxiety, and pain compared to their healthy counterparts. Subjects participated in six one-on-one sessions over a 10-week period with a certified instructor who designed each yoga practice based on a preestablished list of 30 yoga asanas. Questionnaires evaluating pain, sleep disturbance, sustained anxiety, immediate anxiety, and depression were administered. Differences between premeasures and postmeasures were evaluated using a two-sided test. Twenty subjects were assessed (12 females/8 males), median age of 11 (7–20) years. Mean immediate anxiety scores decreased (before session to after session 29 to 23.6, respectively, *p* < 0.001). Joint pain improved (3.25 to 3.65, *p* = 0.028). CFQ-R emotion subscale improved from 79.2 to 85 (*p* = 0.073), and the respiratory subscale improved from 66.7 to 79.2 (*p* = 0.076). Other results were less notable. We conclude that yoga may reduce immediate anxiety and joint pain in patients with CF.

## 1. Introduction

Cystic fibrosis (CF) is the most common genetic, life-limiting, chronic, multiorgan disease among Caucasian populations affecting approximately 30,000 children in the United States and 70,000 worldwide [1, 2]. Although recent advances in diagnosis and treatment have led to increases in lifespan, CF remains one of the most difficult chronic conditions to manage [3, 4] and patients with CF suffer from declining health-related quality of life as the disease progresses [5, 6]. Studies have shown that children with CF experience symptoms of pain, sleep disturbance, anxiety, and depression [7–19].

There is evidence in healthy populations that poor sleep is associated with negative behavior and decreased school performance [20, 21]. Cavanaugh et al. measured sleep quality in fifty subjects with CF (age 6–19 years) using actigraphy. Forty-two participants (84%) experienced poor sleep which was associated with worsening attention and attitude functioning [19]. In addition, there may be an association between decline in pulmonary function and sleep disruption [22] ultimately impacting quality of life for individuals with CF [14].

Anxiety and depression are well-recognized correlates of chronic illness [23–26] and have become an increasing concern in the CF community [4, 10]. These factors may contribute to decreased quality of life as well as decreased adherence to treatments [3, 4, 10, 27]. A meta-analysis evaluating 31 studies and 18,245 subjects with a variety of chronic health diseases revealed subjects with depression were 1.76 times more likely to be nonadherent to their treatment regimens [26].

As other secondary health conditions are increasingly identified in patients with CF, it is important to identify and treat these issues in order to improve quality of life and treatment adherence. Complementary therapy offers a variety of symptom management techniques and may be particularly beneficial for those patients who deal with a complex chronic disease treatment regimen. A study, conducted in subjects with CF, assessed the prevalence of complementary or alternative therapy and demonstrated that 75% of subjects used at least one method of complementary/alternative therapy and described it as being helpful [28].

Yoga has become an increasingly popular complementary therapy to improve and maintain health and well-being. A growing body of research suggests that yoga has significant psychophysiological benefits and clinical relevance as a complementary therapeutic practice [29]. People who participate in yoga may benefit from improved muscle strength, flexibility, blood circulation, oxygen uptake, and hormone function [30]. Research suggests benefits of yoga for various diseases including respiratory disorders, cancers, rheumatoid arthritis, autism spectrum disorder, depression, obesity, diabetes, end stage renal disease, hemophilia, and gastrointestinal disorders [31–55]. Furthermore, it has been shown to improve psychological outcomes in adults with chronic obstructive pulmonary disease [56]. These findings extend to pediatric populations [42–48]. Similarly, evidence supports the efficacy of yoga in adults and children with asthma, showing improvements in quality of life, and, in some cases, improvement in pulmonary function and biochemical profiles, although more high quality research is needed [57–61].

These results set the stage for this study and the goal to explore the possible benefits of yoga in decreasing/alleviating symptoms of pain, sleep disturbance, anxiety, and depression and improving quality of life in children with cystic fibrosis.

## 2. Materials and Methods

### 2.1. Subjects

Subjects were eligible if they were between the ages of 7 and 21 years; had a diagnosis of CF; had no medical contraindications at the time of participation such as advanced liver disease, organ transplant, or severe pulmonary exacerbation; had no participation in an investigational treatment study within 30 days of enrollment; and were not participating in any other yoga practice. Subjects were recruited over the phone or at a clinic visit from both Children's Hospitals and Clinics of Minnesota and University of Minnesota. Subjects were enrolled after obtaining informed consent. The Institutional Review Board of Children's Hospitals and Clinics of Minnesota, Minneapolis, Minnesota, approved this study.

### 2.2. Yoga Certified Instructors and Training

Four certified yoga instructors were selected based on experience with yoga, pediatrics, and chronic disease. Each instructor had a minimum of 200 hours of yoga teacher training from certified yoga alliance schools such as Core Power and Atmananda Yoga Studio. Instructors participated in a two-day training for this study that included CF disease information, consistent use of preselected asanas (postures), administration of questionnaires, session planning/logistics, patient interaction/communication, and the facility/environment.

### 2.3. Intervention

Each subject participated in six private (one-on-one) yoga sessions over a ten-week period.  Table 1 describes the timing intervals of the yoga sessions. Parents were asked to remain in the waiting room during each session. Sessions were spaced at least seven days apart. The ten-week period was selected to allow for flexibility with family schedules. Sessions were one hour in length and included 40 minutes of yoga practice with 10 minutes before and after the intervention to complete questionnaires and discuss the session activities. The same location/room was used for all sessions to reduce possible environmental influences.

To establish standardization, a preestablished list of 30 asanas and associated modifications were selected specifically for this study by the investigators who have also had yoga instructor experience. Each subject's yoga practice was designed from the preestablished list according to their energy levels, attitudes, physical limitations, and competencies on the day of the session. To maximize the impact of each session, instructors were allowed to adjust the patient's practice within the preestablished list as needed. Because yoga typically allows for individualization of this nature, it was viewed as a potentially effective intervention due to the diverse needs of the CF population. Subjects were encouraged but not required to continue the yoga practice at home between sessions.

### 2.4. Questionnaires

The questionnaires (*Cystic Fibrosis Questionnaire-Revised* (CFQ-R),* Memorial Symptom Assessment Scale* (MSAS),* Hospital Anxiety and Depression Scale* (HADS),* Center for Epidemiological Studies-Depression Scale for Children* (CES-DC), and the* Additional Pain Symptoms Assessment* (APSA)) were administered to assess subjects' symptoms of pain, sleep, anxiety, depression, and quality of life immediately before the first session and two weeks after the last session. The* Spielberger State-Trait Anxiety Inventory for Children* (STAIC) questionnaire was administered before and after each yoga session to assess immediate anxiety reduction.  Table 1 describes the timing of questionnaire distribution.

The STAIC is designed to assess both state anxiety, which is how a person feels at the immediate time, and trait anxiety, which describes how likely a person is to react with anxiety to a threatening situation [62]. For this study, we focused on the measure for state anxiety to evaluate a potential change in immediate anxiety levels.

CFQ-R is a disease-specific health-related quality of life questionnaire designed to measure the physical, emotional, and social impact of CF on pediatric patients [63, 64]. The CFQ-R also assesses five subdomains specific to cystic fibrosis: (1) body image, (2) eating disturbances, (3) treatment burden, (4) respiratory symptoms, and (5) digestive symptoms. Three versions of the CFQ-R were used: CFQ-R for ages 6 to 11 years, CFQ-R for ages 12 and 13 years, and CFQ-R for ages 14 to adults [64].

The MSAS assesses multiple symptoms in patients with advanced illnesses. The original tool allows for assessment of 32 symptoms and three dimensions of frequency, severity, and distress. Subscales for analysis include physical distress, psychological distress, and a global distress index [65]. The tool was validated with cancer patients and has been adapted multiple times for use in research with a variety of populations with advanced medical illnesses [66]. A subscale for CF adults was validated for this tool in 2008 with a study that examined symptom prevalence and characteristics in 303 adult patients with CF [67]. Two versions were used for this study: MSAS for ages 7 to 12 and MSAS for ages 10 to 18 [68, 69].

The HADS is a validated 14-item instrument that screens for depression and anxiety in children 12 to 18 years of age who have chronic medical conditions. This validated questionnaire has been used extensively in several countries and is available in different languages [70]. The 14 items are divided into subscales for anxiety and depression, both of which were used for this study. CES-DC is a validated 20-item instrument that evaluates symptoms of depression for ages 12 to 18 [71–73]. This tool was selected in addition to the HADS because it evaluates symptoms of sleep as well as depression. The CES-DC includes three dimensions: behavior, cognition, and happiness.

The APSA is an unvalidated questionnaire developed at our institution in an effort to thoroughly assess pain symptoms that are not captured in other tools. The survey specifically documents abdominal, chest, limb, and joint pain experienced by subjects in the previous two weeks with the frequency of pain rated on a Likert scale of 1–4 (1 = always, 2 = often, 3 = sometimes, and 4 = never) as follows.

*Additional Pain Symptoms Assessment*. In the past two weeks did you have any of the following:

Abdominal Pain?

□ Always
□ Often
□ Sometimes
□ Never

Chest Pain?

□ Always
□ Often
□ Sometimes
□ Never

Limb Pain?

□ Always
□ Often
□ Sometimes
□ Never

Joint Pain?

□ Always
□ Often
□ Sometimes
□ Never

All questionnaires were given to subjects based on the age at enrollment. The MSAS, CES-DC, and HADS were completed by subjects over the validated age range, but not by subjects below the validated age range.  Table 2 describes the validation age and purpose of each questionnaire.

### 2.5. Analytic Approach

Median and range (or mean and standard deviation) were used to describe the continuous variables such as mean STAIC score. The nonparametric Wilcoxon Paired Signed Ranks Test was used to compare before and after scores of anxiety, depression, pain, sleep disturbance levels, and quality of life measured by STAIC, CFQ-R, MSAS, HADS, and CES-DC. A Chi-square test was used to compare the subjects with sleep difficulty before and after yoga sessions. McNemar test was used to compare the binary variables between preintervention and postintervention. All tests employed an alpha level of .05 and were two-sided. We also used the Benjamini-Hochberg Technique (1995) to control the False Discovery Rate across all comparisons in Table 4 [74]. SPSS V15.0 was used to conduct the analyses (SPSS for Windows version 15.0, SPSS Inc., Chicago, IL).

## 3. Results

A total of 21 subjects with mild CF lung disease were enrolled in the study. One subject withdrew and 20 subjects completed the study. Analysis included 8 males and 12 females with a mean age of 11 years (range 7 to 20) and mean FEV1%P (forced expiratory volume in 1 second) of 86%.  Table 3 describes the study population demographics. Nineteen subjects completed six individual yoga sessions and one completed only five sessions due to a CF related respiratory illness. All sessions were completed within a rolling ten-week timeframe. Table 4 shows all the results with notable findings in bold. Only the STAIC remained significant after adjusting for the false positive rate.

**Figure 1:**
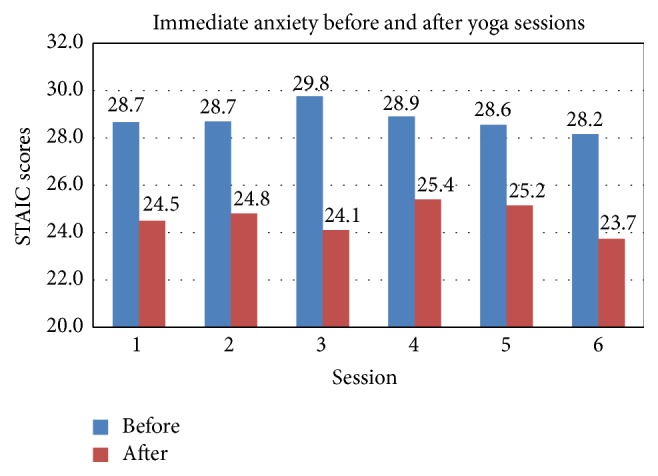
STAIC questionnaire: average mean scores for immediate anxiety reduction before and after yoga sessions: all subjects.

**Figure 2:**
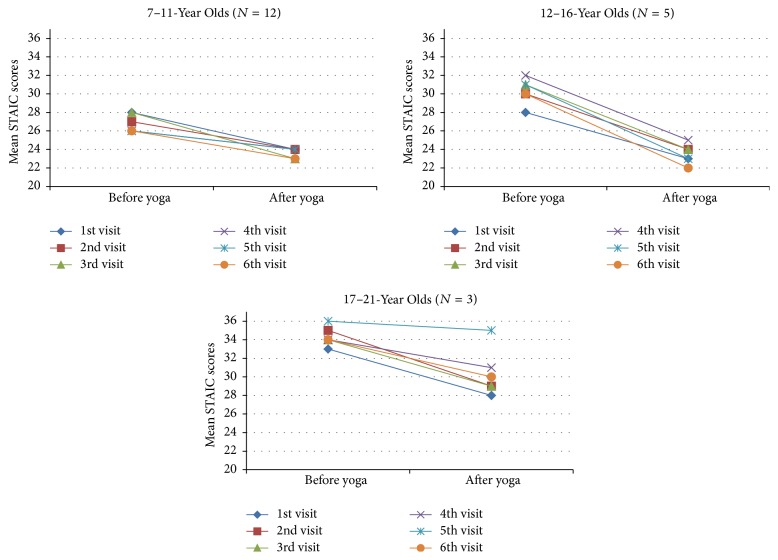
STAIC questionnaire: average mean scores for immediate anxiety reduction before and after yoga sessions: by age groups.

Immediate anxiety improved from before each session (median 29, range 19–39) to after each session (median 23.4, range 20–30) on the STAIC (*p* < 0.001) (Figures 1 and 2). Scores for immediate anxiety also improved when comparing before the first session (median 28, range 19–39) to after the last session (median 26, range 20–31, *p* = 0.024). Sustained anxiety and depression did not show improvement on the CES-DC or HADS.

The CFQ-R showed no notable improvements in quality of life. The domains emotion from before (median 79.2, range 33.3–100) to after (median 85, range 33.3–95.8) yoga intervention (*p* = 0.073) and respiratory before (median 66.7, range 38.9–100) to after (median 79.2, range 38.9–100) yoga intervention (*p* = 0.076) trended towards significance. There were no notable findings for social impact, physical, eating disturbances, body image, social impact, treatment burden, and digestive symptoms.

Pain was evaluated by the MSAS and the APSA. The MSAS showed no notable improvement in self-reported pain. Forty-five percent (*n* = 9) of subjects experienced pain before intervention compared to 35% after (*n* = 7, *p* = 0.625). The APSA showed an improvement in joint pain from a mean score of 3.25 before intervention to 3.65 after intervention on a scale of 1 to 4 with 3 indicating sometimes having joint pain and 4 indicating never having joint pain (*p* = 0.028).

The MSAS showed no notable changes in sleep disturbance with 25% (*n* = 5) of subjects reporting difficulty sleeping before intervention compared to 15% after (*n* = 3, *p* = 0.687). The domain for sleep difficulty before (median 0, range 0–3) to after (median 0, range 0-1) yoga intervention trended towards significance (*p* = 0.088).

There was one adverse event of respiratory illness reported during the study, which was not considered related to the yoga therapy/intervention.

## 4. Discussion

Recent advances in the diagnosis and treatment of CF including universal newborn screening and preventive treatments for complications have led to dramatic improvements in lifespan. As life expectancy improves, researchers are evaluating secondary sequelae including pain, sleep disturbance, anxiety, depression, and decreased quality of life that cause psychological distress [10]. This burden is significant in this patient population and we aimed to assess if therapeutic yoga might help alleviate some of these effects.

A previous study in our center assessing symptoms among 39 subjects with CF, ages 7 to 18 years, found that 72% of subjects experienced depression, 16% expressed problems with sleep, 37% experienced moderate to severe long-term anxiety, and 29% complained of pain (abdomen, chest, limbs, and joints) [11]. A similar study evaluating pain in a group of 46 subjects with CF, ages 8 to 18 years, found that 50% of subjects experienced stomach pain, 37% chest pain, and 33% head pain [7].

After systematic review [75, 76] we found only one prior study performed by Ruddy et al. to evaluate yoga as a complementary therapeutic practice in children with cystic fibrosis [76]. The study presented here is the largest to date and the first with a primary aim is to evaluate therapeutic benefit; however, this is a pilot study and the results should be considered suggestive but not conclusive. The other study was a prospective pilot study conducted primarily to evaluate the safety of a standardized yoga program for patients with cystic fibrosis. The mean (SD) CFQ-R respiratory domain score increased significantly from screening to end of study, 67.9 (11.40 to 82.1 (9.9)), (*p* = 0.04) in the 10 participants who completed the program. No other outcome measures reached significance and it was recommended that further testing with larger trials be conducted [76]. Twenty participants completed the yoga program offered at our site. As with Ruddy et al. [76], the subjects in our study tolerated the yoga therapy safely.

Our most notable finding was an improvement in immediate anxiety from before each yoga therapy session (median 29, range 19–39) to after (median 23.4, range 20–30) on the STAIC (*p* < 0.001). Prior to study participation immediate anxiety levels for the study population were within normal limits when compared to the general population for this age group. However, STAIC scores trended towards improvement from immediately before a single yoga session to immediately after a session as shown in Figure 1. Additionally, the first questionnaire completed at enrollment compared to the last questionnaire at the follow-up visit showed a significant decrease in immediate anxiety (*p* = 0.024). Increased anxiety levels may impair attention and disrupt cognitive information processing [77], so an intervention that is able to reduce immediate anxiety may provide real benefit. One subject expressed how, when feeling anxious at school, she practiced a breathing technique that she learned from her yoga instructor and found the method to be helpful. Unfortunately, we were not able to show any notable improvement in sustained anxiety or depression.

The APSA showed a notable improvement in reported joint pain from prior to initiating yoga therapy to after the conclusion of yoga sessions (*p* = 0.028). The MSAS, a tool that measures more generalized self-reported pain, decreased from 45% of subjects experiencing pain before treatment to 35% after but, with the admitted limitation of our small sample size, was not notable (*p* = 0.625). Pain is significant in patients with CF [7, 11] and it is a symptom that people with CF of all ages experience with an increase in severity and duration with age, unrelated to disease severity [77, 78]. Yoga may be helpful for managing at least some of these pain symptoms. Beginning the practice and lifestyle of yoga early in life may provide larger benefits later in life.

The CFQ-R emotion subscale, which addressed feelings of worry, loneliness, sadness, and the desire to make future plans, did not show notable improvements (*p* = 0.073). The respiratory subscale also did not show notable improvements (*p* = 0.076) in our study. It is worth noting that the Ruddy et al. study did show a significant improvement in the CFQ-R respiratory subscale in their ten-participant cohort (*p* = 0.04) with a more intense session period (twice a week for 10 weeks), but they were also unable to show significant improvement in the emotion subscale (*p* = 0.13) The Ruddy et al. protocol was to practice yoga twice a week for 8 weeks and had a mean attendance of 14 sessions [76]. Larger controlled trials may be warranted to further investigate yoga and quality of life improvements in patients with CF, especially since yoga has been shown to increase quality of life scores in other chronic disease populations including breast cancer, rheumatoid arthritis, and children with hemophilia [40, 42, 51] as well as respiratory diseases such as asthma [79].

The study presented here did not show notable improvement in sleep difficulties with yoga therapy but did demonstrate a trend towards significance in sleep difficulty before (median 0, range 0–3) to after (median 0, range 0-1) yoga intervention (*p* = 0.088). The prevalence of sleep difficulties is well documented in the CF population [8, 11–19] with possible etiologies including nocturnal hypoxemia preventing REM sleep [12, 13] and nocturnal cough.

### 4.1. Limitations

Our study demonstrated two promising and notable outcomes of reduction in pain and immediate anxiety which suggest a trend towards improvement despite the low power of our 20-participant cohort. Demonstrating significance in this small, healthier CF cohort may have been a limitation since our population has excellent pulmonary outcomes overall. Thus, a lower pain prevalence and symptomatic burden within our population is expected. This study lacked a control group, which may have strengthened our findings. We did not assess the duration of the short-term anxiety relief experienced. While this study demonstrated an immediate improvement in anxiety, we do not know if this relief lasts minutes, hours, or days. To this end, questionnaires assessing general anxiety administered during the yoga therapy period would be of interest. We did not have a validated tool for patients with CF to assess localized pain such as limb and joint.

Our study was limited by the small number of yoga sessions: 6 sessions over 10 weeks for 19 participants and 5 sessions for 1 participant. It is possible that the intensity and duration of the yoga therapy in our study were too small, with too few yoga sessions spread too far apart over too short a time. Larger studies with an objective measurement of sleep quality, such as with sleep actigraphy, during an experimental yoga therapy trial may be beneficial.

## 5. Conclusions

This yoga session interventional pilot study for patients with CF demonstrated a notable decrease in immediate anxiety from prior to and just after yoga therapy sessions. This study also showed a notable decrease in reported joint pain from before the trial of yoga therapy began to after it was completed. Yoga as a complementary therapy may be a valuable option for patients with CF who are experiencing pain or anxiety related to their health, school, or social activities. Our results are consistent with the literature that yoga is safe and well tolerated in children and adolescents with mild to moderate lung disease. Our findings of notable improvements in a small pilot study with a relatively low dose of yoga therapy and other measures trending towards significance suggest that larger controlled trials conducted to determine the benefits of yoga for patients with CF would be valuable.

## Figures and Tables

**Table 1:** Study schema: timing intervals of yoga sessions, questionnaire administration.

	Week	Week	Week	Week	Week	Week	Week	Week
	1	2	3	4	5	6–10^*∗*^	+1	+2
Yoga session	X	X	X	X	X	X		
STAIC	Before	Before	Before	Before	Before	Before		X
	After	After	After	After	After	After		
CFQ-R	X							X
MSAS	X							X
HADS	X							X
CES-DC	X							X
APSA	X							X

^*∗*^Participants were allowed 10 weeks to complete 6 therapeutic yoga sessions.

(i) Before: test administered immediately prior to yoga session.

(ii) After: test administered immediately after yoga session.

(iii) X indicates the week a test was conducted.

**Table 2:** Questionnaires: age validation and purpose.

Tools	Validation	Purpose
STAIC	6–17 years of age	Anxiety
STAIC	18+ years of age	Anxiety
CFQ-R	6–14+ years of age	Quality of life
MSAS^*∗*^	7–18 years of age	Pain and sleep disturbance
CES-DC^*∗*^	12–18 years of age	Depression
HADS^*∗*^	12–18 years of age	Anxiety and depression
APSA	Not validated	Pain

^*∗*^The MSAS, CES-DC, and HADS were completed by subjects over the validated age range but not by subjects below the validated age range.

**Table 3:** Demographics Data.

Study ID	Gender	Ethnicity	Age	Baseline FEV1%P^*∗*^	Lung disease Severity^*∗∗*^
001	F	Caucasian	14.7	86%	Normal
002	F	Not answered	13.1	116%	Normal
003	F	Not answered	14.7	71%	Mild
005	M	Caucasian	12.1	96%	Normal
006	M	Caucasian	11.9	82%	Normal
007	F	African American	17.2	82%	Normal
008	F	Caucasian	9.7	57%	Moderate
009	M	Caucasian	9.0	83%	Normal
010	F	Hispanic	14.0	66%	Mild
011	M	Caucasian	11.9	95%	Normal
012	F	Caucasian	19.5	94%	Normal
013	F	Caucasian	8.7	116%	Normal
014	M	Caucasian	10.0	89%	Normal
015	M	Caucasian	7.6	102%	Normal
016	M	Caucasian	7.6	74%	Mild
017	F	Caucasian	19.4	74%	Mild
018	F	Caucasian	8.9	75%	Mild
019	F	Caucasian	7.1	52%	Moderate
020	F	Caucasian	7.3	111%	Normal
021	M	Caucasian	7.3	106%	Normal

^*∗*^FEV1%P is the forced expiratory volume in 1 second.

^*∗∗*^Lung disease severity measured by FEV1%P: normal is ≥80%, mild is 60%–79%, moderate is 40–59, and severe is ≤40%.

**Table 4:** Results of 6 Yoga sessions over a ten-week intervention in children with cystic fibrosis.

Measure	*N*	Before intervention	After intervention	*p* value
STAIC, median (range)	20	28 (19, 39)	26 (20, 31)	0.024^*∗*^

CFQ-R, median (range)				
Emotion		79.2 (33.3, 100)	85 (33.3, 95.8)	**0.073**
Physical		83.3 (54.2, 100)	85.4 (45.8, 100)	0.924
Social impact		84.5 (47.6, 95.2)	76.2 (42.9, 95.2)	0.393
CF specific domain				
Body image		83.3 (22.2, 100)	88.9 (33.3, 100)	0.287
Respiratory symptoms		66.7 (38.9, 100)	79.2 (38.9, 100)	**0.076**
Digestive symptoms		66.7 (33.3, 100)	72.2 (33.3, 100)	0.748
Treatment burden		55.6 (22.2, 100)	66.7 (0, 100)	0.629
Eating disturbances	20	94.4 (22.2, 100)	88.9 (22.2, 100)	0.721

MSAS				
Pain, *n* (%)		9 (45)	7 (35)	0.625
Sleep difficulty, *n* (%)		5 (25)	3 (15)	0.687
Sleep difficulty, median (range)	20	0 (0, 3)	0 (0, 1)	**0.088**

HADS, median (range)				
Anxiety		8.5 (0, 15)	9 (1, 15)	0.497
Depression	10	6.5 (0, 12)	8 (0, 12)	0.491

CES-DC, median (range)	10	16.5 (0, 28)	16 (10, 42)	0.141

APSA				
Abdominal pain, median (range)		4 (1, 4)	3.5 (1, 4)	0.564
Chest pain, median (range)		4 (3, 4)	4 (2, 4)	0.480
Limb pain, median (range)		4 (2, 4)	4 (2, 4)	0.248
Joint pain, median (range)		4 (1, 4)	4 (2,4)	0.033^*∗*^
Joint pain, mean (95% CI)	20	3.3 (2.8, 3.7)	3.7 (3.4, 3.9)	0.028^*∗*^

		*Before each session*	*After each session*	

STAIC, median (range)	20	29.1 (19, 39)	23.4 (20, 30)	<**0.001** ^*∗∗*^

^*∗*^Significantly different at *α* = 0.05.

^*∗∗*^Significantly different at *α* = 0.05 adjusting for 18 comparisons (only STAIC remained significant after adjustment for multiple variables).

STAIC: immediate anxiety. Total score ranging from 0 to 60, lower score is better.

CFQ-R: scaled score between 0 and 100, higher is better.

MSAS: scaled score between 0–4 for each domain, lower is better.

HADS: scaled score between 0 and 21, lower is better.

CES-DC: scaled score between 0 and 60, lower is better.

APSA: scale of 1 to 4, higher is better.
